# Associations among internet addiction, lifestyle behaviors, and dental caries among high school students in Southwest Japan

**DOI:** 10.1038/s41598-022-22364-0

**Published:** 2022-10-15

**Authors:** Masanori Iwasaki, Satoko Kakuta, Toshihiro Ansai

**Affiliations:** 1 Research Team for Promoting Independence and Mental Health, Tokyo Metropolitan Institute of Gerontology, 35-2 Sakae-Cho, Itabashi-Ku, Tokyo, 173-0015 Japan; 2 Division of Community Oral Health Development, Kyushu Dental University, 2-6-1 Manazuru, Kokurakita-Ku, Kitakyushu, 803-8580 Japan

**Keywords:** Dental diseases, Epidemiology

## Abstract

Internet addiction (IA) negatively affects adolescents’ lifestyle behaviors. Inappropriate lifestyle behaviors could have negative effects on dental health. This cross-sectional study aimed to test whether IA was indirectly associated with dental caries through unhealthy lifestyle behaviors among high school students in southwest Japan. IA was characterized by a Young's Internet Addiction Test score of ≥ 50, unhealthy lifestyle behaviors by a cumulative count of 8 different lifestyle behaviors (termed the unhealthy lifestyle behavior index [ULBI]), and dental caries by the number of decayed, missing, and filled permanent teeth (DMFT). Poisson regression and linear regression models were fitted to the relationship, with IA as the exposure, the ULBI as the mediator, and the DMFT as the outcome. The natural indirect effect (NIE) and the proportion mediated by the ULBI were estimated by performing a mediation analysis. Overall, 1562 high school students were included. IA was observed in 406 participants and was associated with a larger DMFT. The ULBI significantly mediated the association between IA and the DMFT (NIE: incidence rate ratio = 1.05, 95% confidence interval = 1.03–1.07, proportion mediated = 64.3%). Dental caries was more common in our cohort of high school students with IA, which is partially explained by these students having unhealthy lifestyle behaviors.

## Introduction

Currently, the internet is an essential part of our daily lives. It is a useful tool allowing us to access unlimited information. However, there is a problem related to internet use. Excessive or poorly controlled preoccupations, urges or behaviors regarding internet use, termed internet addiction (IA)^1^, have been demonstrated to be associated with depression, aggressive behaviors, psychiatric symptoms, and interpersonal problems among adolescents^2–4^. Furthermore, IA is considered a serious behavioral problem because of its adverse effect on several lifestyle-related factors. IA can result in physical inactivity, short sleep duration, and inappropriate dietary habits, such as irregular mealtimes and frequent consumption of soft drinks and snacks^5,6^.

Dental caries is a common chronic disease in adolescents^7^ and is associated with pain, decreased appetite, difficulty eating, malnutrition, poor school performance and attendance, poor quality of life, and future tooth loss^8–10^. Dental caries is a result of bacterial activity in dental plaque. The acid produced by the oral bacterial fermentation of dietary carbohydrates in dental plaque causes tooth demineralization, resulting in tooth decay^11^. Several lifestyle behaviors are considered risk factors for dental caries because they disrupt good oral hygiene and are associated with bacterial activity and plaque accumulation. Previous studies have demonstrated that less frequent toothbrushing, irregular meal frequency, and higher consumption of snacks and sweetened drinks are associated with dental caries^11–14^.

Based on the evidence indicating that IA negatively affects lifestyle behaviors and that inappropriate lifestyle behaviors have adverse effects on dental caries, IA could be indirectly associated with dental caries through unhealthy lifestyle behaviors among adolescents. To date, there is a lack of knowledge on the dental health of adolescents with IA. We therefore conducted a cross-sectional study with the aim of examining the interrelationships among IA, lifestyle behaviors, and dental caries in high school students in southwest Japan. Our null hypothesis was that IA does not have an indirect association with dental caries via unhealthy lifestyle behaviors among this population.

## Results

### Study population

Of all 1706 students (722 10th grade, 554 11th grade, and 430 12th-grade students; 929 male and 777 female), 1691 students had health checkup data at the school in the fiscal year 2019. Among those 1691 students, 1635 students (96.7%) agreed to participate in our study. Of those students, 73 (21 who were diagnosed by physicians as having medical conditions or abnormalities requiring close examination and 52 who had incomplete data) were excluded, resulting in the study population being composed of 1562 students (676 10th grade, 503 11th grade, and 383 12th-grade students; 850 male and 712 female).

As presented in Table 1, 406 (26.0%) out of the 1562 study participants were classified as suffering from IA. Individual IAT items and the study participants' responses to these items are summarized in Supplementary Table S1. The participants with IA had a larger number of decayed, missing, and filled permanent teeth (DMFT) (*p* < 0.01); had a larger unhealthy lifestyle behavior index (ULBI) (*p* < 0.01); were more likely to be in the 11th grade (*p* = 0.03); were more likely to be female (*p* = 0.01); and were less likely to use fluoride toothpaste (*p* = 0.02). IA was associated with all of the components of the ULBI (*p* < 0.05) except for interdental cleaning device use.

**Table 1:** Characteristics of the study population according to the presence of internet addiction (N = 1562).

		Internet addiction		
	Total	No	Yes	*p* value*
	N = 1562	N = 1156	N = 406	
IAT score^†^	41.9 (13.4)	35.6 (7.9)	59.8 (8.6)	< 0.01
DMFT^‡^	1 (0–3)	0 (0–3)	1 (0–3)	0.049
DT^‡^	0 (0–0)	0 (0–0)	0 (0–0)	0.35
**School grade**^**§**^				0.03
10th	676 (43.3%)	519 (44.9%)	157 (38.7%)	
11th	503 (32.2%)	352 (30.4%)	151 (37.2%)	
12th	383 (24.5%)	285 (24.7%)	98 (24.1%)	
**Sex**^**§**^				0.01
Male	850 (54.4%)	651 (56.3%)	199 (49.0%)	
Female	712 (45.6%)	505 (43.7%)	207 (51.0%)	
ULBI^‡^	2 (1–3)	2 (1–3)	3 (2–4)	< 0.01
**Components of the ULBI**^**§**^				
Brushing teeth < 2 times/day	162 (10.4%)	106 (9.2%)	56 (13.8%)	0.01
Not brushing teeth before bed	447 (28.6%)	302 (26.1%)	145 (35.7%)	< 0.01
Not using interdental cleaning devices	976 (62.5%)	710 (61.4%)	266 (65.5%)	0.14
Sleep duration < 6 h/day	358 (22.9%)	218 (18.9%)	140 (34.5%)	< 0.01
One or more regular soft drinks per day	273 (17.5%)	185 (16.0%)	88 (21.7%)	0.01
One or more regular sweet snacks per day	321 (20.6%)	209 (18.1%)	112 (27.6%)	< 0.01
Irregular dinner consumption	1057 (67.7%)	743 (64.3%)	314 (77.3%)	< 0.01
Late-evening snacking ≥ 5 days per week	111 (7.1%)	73 (6.3%)	38 (9.4%)	0.04
Receiving professional tooth brushing instruction^§^	910 (58.3%)	662 (57.3%)	248 (61.1%)	0.18
Use of fluoride toothpaste^§^	811 (51.9%)	621 (53.7%)	190 (46.8%)	0.02
Fluoride application^§¶^	970 (62.1%)	721 (62.4%)	249 (61.3%)	0.71
Regular dental visits^§^	675 (43.2%)	496 (42.9%)	179 (44.1%)	0.68
Overweight^§^	139 (8.9%)	103 (8.9%)	36 (8.9%)	0.98

*IAT* Young’s Internet Addiction Test, *DMFT* number of decayed, missing, and filled permanent teeth, *IQR* interquartile range, *SD* standard deviation, *ULBI* unhealthy lifestyle behavior index.

Underlined text indicates data with significant adjusted standardized residuals.

*Comparison between groups.

^†^Presented as the mean (SD).

^‡^Presented as the median (IQR).

^§^Presented as n (%).

^¶^Either a professional topical fluoride application or fluoride mouth rinse.

### Crude associations of dental caries with IA, unhealthy lifestyle behaviors, and other factors

Table 2 shows the results of Poisson regression analyses. IA and the ULBI were associated with higher IRRs for the DMFT (IRR 1.12, 95% CI 1.03–1.21, *p* < 0.01 for IA and IRR 1.10, 95% CI 1.07–1.13, *p* < 0.01 for ULBI, respectively). Receiving tooth brushing instruction (IRR 1.38, 95% CI 1.28–1.48, *p* < 0.01) and regular dental visits (IRR 1.21, 95% CI 1.12–1.29, *p* < 0.01) were associated with a higher IRR for the DMFT. The experience of fluoride application was associated with a lower IRR for the DMFT (IRR 0.90, 95% CI 0.83–0.96, *p* < 0.01).

**Table 2:** Poisson regression analysis for the crude associations of dental caries with internet addiction, the unhealthy lifestyle behavior index, and other factors (N = 1562).

Variables	Outcome = DMFT		
	IRR	95% CI	*p* value*
Internet addiction	1.12	1.03–1.21	< 0.01
ULBI (per one-point increase)	1.10	1.07–1.13	< 0.01
**School grade**			
10th	Reference		
11th	1.41	1.29–1.53	< 0.01
12th	1.71	1.56–1.86	< 0.01
Male (vs. female)	0.96	0.89–1.03	0.25
Receiving professional tooth brushing instruction	1.38	1.28–1.48	< 0.01
Use of fluoride toothpaste	0.95	0.89–1.02	0.18
Experience of fluoride application^†^	0.90	0.83–0.96	< 0.01
Regular dental visits	1.21	1.12–1.29	< 0.01
Overweight	0.99	0.88–1.12	0.90

*CI* confidence interval, *DMFT* number of decayed, missing, and filled permanent teeth, *IRR* incidence rate ratio, *ULBI* unhealthy lifestyle behavior index.

*Except for unhealthy lifestyle score, grade, and sex, IRRs and CIs of being positive are presented.

^†^Either a professional topical fluoride application or fluoride mouth rinse.

### Interrelationships among IA, unhealthy lifestyle behaviors, and dental caries

Table 3 shows the results of the mediation analysis. The null hypothesis that there were no associations among IA, unhealthy lifestyle behaviors, and dental caries was rejected. The ULBI significantly mediated the association between IA and the DMFT (NIE: IRR 1.05, 95% CI 1.03–1.07, *p* < 0.01, proportion mediated = 64.3%). The natural direct effect and total effect of IA on the DMFT were represented by IRRs of 1.03 (95% CI 0.95–1.12, *p* = 0.48) and 1.08 (95% CI 0.9999–1.17, *p* = 0.05), respectively. When different cutoff points of the IAT were used to classify the students with IA, the mediation of the ULBI remained significant (NIE: IRR 1.04, 95% CI 1.01–1.07, *p* < 0.01, proportion mediated = 24.8%; Supplementary Table S2). These results are consistent with those of the main analysis presented in Table 3. In addition, IA was significantly indirectly associated with DT through the ULBI (NIE: IRR 1.18, 95% CI 1.08–1.30, *p* < 0.01; Supplementary Table S3).

**Table 3:** Mediation of the associations between internet addiction and dental caries by unhealthy lifestyle behaviors (N = 1562).

	Outcome = DMFT		
	Mediator = ULBI		
Exposure = Internet Addiction (1 = present [IAT score ≥ 50], 0 = absent [IAT score < 50])	IRR*	95% CI	*p* value
Natural direct effect	1.03	0.95–1.12	0.48
Natural indirect effect	1.05	1.03–1.07	< 0.01
Total effect	1.08	0.9999–1.17	0.05
Proportion mediated	64.3%		

*CI* confidence interval, *DMFT* number of decayed, missing, and filled permanent teeth, *IAT* Young’s Internet Addiction Test, *IRR* incidence rate ratio, *ULBI* unhealthy lifestyle behavior index.

*Adjusted for grade, sex, receiving professional tooth brushing instruction, use of fluoride toothpaste, fluoride application, regular dental visits, and overweight.

## Discussion

In this cross-sectional study involving high school students in southwest Japan, we investigated the interrelationships among IA, lifestyle behaviors, and dental caries. Our mediation analysis rejected the null hypothesis that there were no associations among IA, unhealthy lifestyle behaviors, and dental caries. IA indirectly affected dental caries through unhealthy lifestyle behavior.

In this study, we found that IA was associated with short sleep duration, frequent consumption of soft drinks and sweet snacks, irregular mealtimes, and late-evening snacking. These findings are in agreement with those from previous studies investigating IA and lifestyle behaviors^5,6^. In addition, we found that the study participants with IA had poorer oral health behaviors, such as less frequent toothbrushing and not brushing teeth before bed. Previous studies reported that adolescents suffering from IA tend to restrict their eating habits to accommodate their heavy internet use^5,6^. Similarly, those suffering from IA may dedicate less time to oral health behavior so that they can spend more time online. Tooth brushing has less priority and will be missed in that situation. Our study results indicate that IA is a barrier to maintaining good oral hygiene. Poor oral hygiene accelerates dental plaque accumulation and bacterial activity in dental plaque^11–13^, which results in dental caries development and progression. Overall, one potential reason for the higher prevalence of dental caries among the study participants who suffered from IA was the loss of dental plaque control, which was associated with unhealthy lifestyle behavior.

We observed that receiving tooth brushing instruction and regular dental visits were associated with the prevalence and experience of dental caries in this study population, which was contrary to the assumption that appropriate oral health knowledge and skills would be obtained through tooth brushing instruction during dental visits. One potential explanation is that the students who were identified as having dental caries at any health checkup prior to the current study visited dental clinics and received tooth brushing instruction.

The IAT is the most frequently used diagnostic instrument for IA^15^. An IAT cutoff of 40 was suggested to be too low to screen for possible IA in Japanese adolescents^15^. An IAT of 50 was alternatively proposed as the cutoff point^15^. In addition, the IAT of 50 has been used in previous studies conducted in other countries, including China, Italy, and Turkey^16–18^. We therefore chose to use the IAT cutoff point of 50 in this study. When we used an IAT of 40 to classify a participant as having possible IA or no IA in the sensitivity analysis, we still observed that IA indirectly affected dental caries. However, the magnitude of the indirect effect through unhealthy lifestyle behavior was decreased, suggesting that the IAT cutoff point of 40 was not appropriate for differentiating the participants with and without IAT in this study.

The average IAT score of our cohort of high school students was 41.9 (standard deviation = 13.4), which is similar to that of other surveys involving Japanese high school students (average = 45.2, standard deviation = 12.6)^15^. The mean DMFTs in the 10th-, 11th-, and 12th-grade students in this study were 1.51, 2.13, and 2.56, respectively, which were similar to those reported by the Fukuoka Association of School Dentists (i.e., 1.56, 1.90, and 2.21, respectively)^19^. However, it should be noted that this study was conducted in one high school in Fukuoka Prefecture, Japan. The prevalence of IA may depend on the social and cultural background where studies are carried out. In addition, there are inequalities in dental caries prevalence among prefectures in Japan^20^. Overall, it is not clear whether the current findings can be applied to other areas in Japan and the broader population. Further studies are needed to test the generalizability of our results.

The main outcome, the DMFT, represents dental caries prevalence and experience in permanent dentition. Although the DMFT is an important index, it does not always reflect the current cariological situation and need for dental treatment. The DMFT might be reached before IA is established. We also test the interrelationships among IA, unhealthy lifestyle behaviors, and DT. DT represents the presence of untreated caries lesions in the dentition. We found that IA was indirectly associated with a larger DT thorough a larger ULBI. These findings support the results of the main analysis in which the DMFT was set as an outcome. However, it should be noted that this study had a cross-sectional design, limiting our ability to establish temporality. Future longitudinal studies assessing the effect of IA on dental caries incidence are necessary.

This study has several other limitations. First, the ULBI used in this study did not include all possible lifestyle factors. Although each factor has been studied in terms of its association with dental caries and/or IA^5,6,12,13,21^, other factors, such as tooth brushing duration and smoking^5^, are also possibly associated with disease risk. Second, the IAT and ULBI were based on self-reports and therefore were subject to recall bias and misclassification. Finally, as with other mediation analyses, our mediation analyses relied on the counterfactual approach being based on the assumption that there is no unmeasured confounding of the exposure–mediator, mediator–outcome, and exposure–outcome relationships and that there is no exposure-induced mediator–outcome confounding^22^. Even though our analyses were adjusted for the various potential confounding variables evaluated, there might have been unmeasured confounding factors, such as socioeconomic status.

## Conclusion

Our study of high school students in southwest Japan demonstrated that dental caries was more common in students with IA, which is partially explained by the fact that these students had unhealthy lifestyle behaviors.

## Methods

### Study design, setting, and population

This study had a cross-sectional design. The study population was composed of a cohort of 10th–12th-grade students in one high school in Fukuoka Prefecture that is located in the southwest of Japan. On 11 April 2019, they participated in routine medical and dental health checkups at the school. In Japan, students undergo periodic health checkups, as mandated by the School Health and Safety Act. On the same day, a questionnaire regarding internet use and health behavior was administered. Data obtained from health checkups and questionnaires were used for the study.

The inclusion criteria were as follows: at least 15 years of age, able to read and understand Japanese, and had periodic health checkup records at school. The exclusion criteria were as follows: those who were diagnosed by physicians as having medical conditions or abnormalities requiring close examination or who had incomplete data. This study was designed as a part of a student welfare effort. No sample size calculation was performed prior to the study. All the students who met the criteria for inclusion were invited to participate in our study.

This study was conducted in full accordance with the ethics principles of the Declaration of Helsinki and was approved by the Ethics Committee of Kyushu Dental University (approval number: 18–63, approval date: 26 March 2019). Written informed assent from all participants and informed consent from all their guardians were obtained.

### Measure of IA

Data on the level of IA were obtained via a self-administered questionnaire. The Japanese version of Young's Internet Addiction Test (IAT)^23–25^ was used to assess the level of IA. The IAT consists of 20 items regarding internet overuse (Supplementary Table S1). Each item is rated on a 5-point Likert scale ranging from 1 (rarely) to 5 (always). The total score ranges from 20 to 100. According to previous studies^15–18^, a cutoff point of 50 was used to classify a participant as suffering from IA. The reliability and validity of the Japanese version of the IAT have been demonstrated^23,24^. The internal consistency obtained in the study population was demonstrated to be acceptable (Cronbach’s α = 0.91).

### Measure of lifestyle behavior

Eight different lifestyle behaviors (tooth brushing frequency, tooth brushing before bed, interdental cleaning device use, sleep duration, soft drink consumption, sweet snack consumption, meal regularity in the evening, and late-evening snacking), based on a priori knowledge and the assumption of factors related to dental caries and IA^5,6,12–14,21^, were assessed via a self-administered questionnaire. For each lifestyle behavior, a score of 1 was assigned if the behavior was considered unhealthy; otherwise, a score of 0 was assigned. The ULBI was generated by adding the individual points for each of the eight factors and hence ranged from 0 (most healthy) to 8 (least healthy). Specifications for each of the 8 lifestyle behaviors are summarized in Table 4.

**Table 4:** Unhealthy lifestyle behavior index construction.

Components	Survey questions	Possible answer choices	Score allocation
Tooth brushing frequency	How many times a day do you brush your teeth?	2 times/≥ 3 times per day	0
		0 times/1 time per day	1
Tooth brushing before bed	How many days a week do you fall asleep at night without brushing your teeth?	0 days	0
		1–2 days/3–4 days/5–6 days/7 days per week	1
Interdental cleaning device use	Do you use an interdental cleaning device?	Always/sometimes	0
		Do not use	1
Sleep duration	During the past month, how many hours of actual sleep did you get at night?	≥ 6 h	0
		< 6 h	1
Soft drink consumption	How often do you drink sugar-sweetened soft drinks?	< 1 time/1–2 times/3–4 times/5–6 times per week	0
		1 time/2–3 times/4–6 times/7–9 times/ ≥ 10 times per day	1
Sweet snack consumption	How often do you eat sweet snacks?	< 1 time/1–2 times/3–4 times/5–6 times per week	0
		1 time/2–3 times/4–6 times/7–9 times/ ≥ 10 times per day	1
Meal regularity in the evening	Do you eat dinner every day?	Eat at a regular time every day	0
		No set time, but eat every day/Skip a dinner sometimes/Skip a dinner often/Do not eat	1
Late-evening snacking	How many days a week do you have late-evening snacks?	0 days/1–2 days/3–4 days per week	0
		5–6 days/7 days per week	1

### Examination of dental health

Twelve dentists who were blinded to the questionnaire responses were involved in dental health checkups at school. The dentists and students sat facing each other in a knee-to-knee position during the checkup. The status of all erupted permanent teeth was assessed using a graduated mouth mirror (Clear-Mouth Mirrors, FEED, Yokohama, Japan) under sufficient artificial illumination. Tooth status was categorized as sound, decayed, filled, or missing. In accordance with the protocol proposed by the Japan Association of School Dentists^26^, teeth with obvious caries lesions detected on visual clinical examination were categorized as decayed teeth. Then, the number of decayed teeth (DT) and DMFT were determined. Dental radiography was not performed.

All examiners received 1 h of instruction from the author (M.I.) at Kyushu Dental University regarding the appropriate tooth status assessment method (dentist and student position during assessment and diagnostic criteria^26^). Then the pre-study calibration was conducted by examining volunteer patients. All the examiners obtained intraclass correlation coefficients of ≥ 0.92 for DMFT and intra-examiner kappa values of ≥ 0.87 for DT.

### Data collection for covariates

Data on receiving professional tooth brushing instruction (yes or no), use of fluoride toothpaste (yes or no/do not know), fluoride application (either professional topical fluoride application or fluoride mouth rinse) (yes or no/do not know), and regular dental visits (yes or no) were obtained through the self-administered questionnaire. Regular dental visits were confirmed if the participants answered yes to the question, “Do you have regular dental checkups at least once a year?” Data on school grade, sex, physician diagnosis of heart disease, the results of chest X-ray examination and urine examination, and body mass index (BMI) were obtained from the health checkup records. Overweight was defined as a BMI ≥ 25 kg/m^2^.

### Statistical analyses

First, the study population characteristics were described according to the presence of IA. The Shapiro–Wilk test was used to determine whether the continuous variables were normally distributed. Student's *t test*, the Wilcoxon rank-sum test, and the chi-squared test were used, as appropriate. If the overall test for the variables with ≥ 3 categories was significant, post hoc comparisons were performed.

Next, Poisson regression analyses were performed to assess the crude associations of dental caries, expressed as the DMFT, with IA, the ULBI, and other factors among the study participants.

Finally, mediation analysis with a two-way decomposition approach was employed, while setting IA as the exposure, the DMFT as the outcome, and the ULBI as the mediator. A Poisson regression model was specified to be fitted for the outcome variable and a linear regression model to be fitted for the mediator variable. Our null hypothesis was that either or both of the IA-ULBI and ULBI-DMFT associations was zero. The total effect was separated into the natural direct effect, which is the effect of the exposure not explained by the mediator, and the natural indirect effect (NIE), which is the effect of the exposure explained by the mediator and the interaction between the exposure and the mediator. The presence of an exposure-mediator interaction was allowed. The estimates are presented as incidence rate ratios (IRRs) with 95% confidence intervals (CIs). The CIs were derived by bootstrapping based on 1000 replications. School grade, sex, overweight, receiving tooth brushing instruction, use of fluoride toothpaste, fluoride application, and regular dental visits were considered covariates.

To differentiate “no IA” and “possible IA or IA,” other studies have used an IAT score of 40 as the cutoff^3,4^. Therefore, a sensitivity analysis was performed in which a dichotomized IA variable with an IAT cutoff point of 40 was set as the exposure instead of the IA variable with an IAT cutoff point of 50. In addition, another sensitivity analysis was performed in which the DT was set as the outcome instead of the DMFT.

All analyses were performed with statistical software (STATA version 17.0; Stata, College Station, TX, US). The level of significance (two-tailed) was set at 0.05.

## Supplementary Information

Supplementary Information.

## Data Availability

The data are not publicly available due to ethical and legal restrictions imposed by the Ethics Committee of Kyushu Dental University (the data contain potentially identifying or sensitive patient information). Data may be made available from the corresponding author upon request.
