# Supplementary material for: Associations among internet addiction, lifestyle behaviors, and dental caries among high school students in Southwest Japan
Source: Sci Rep. 2022 Oct 15;12:17342. doi: 10.1038/s41598-022-22364-0 (PMC9569321; doi:10.1038/s41598-022-22364-0)
Supplement: Supplementary file 1 — Supplementary Information. [file 41598_2022_22364_MOESM1_ESM.docx]

**Supplemental Information**

**Associations among Internet Addiction, Lifestyle Behaviors, and Dental Caries among High School Students in Japan**

Masanori Iwasaki^1*^, Satoko Kakuta^2^, Toshihiro Ansai^2^

^1^Research Team for Promoting Independence and Mental Health, Tokyo Metropolitan Institute of Gerontology, 35-2 Sakae-cho, Itabashi-Ku, Tokyo 173-0015, Japan

^2^Division of Community Oral Health Development, Kyushu Dental University, 2-6-1 Manazuru, Kokurakita-ku, Kitakyushu 803-8580, Japan

***Corresponding Author**

Masanori Iwasaki

Research Team for Promoting Independence and Mental Health, Tokyo Metropolitan Institute of Gerontology, 35-2 Sakae-cho, Itabashi-Ku, Tokyo 173-0015, Japan

Tel.: +81 33 964 3241 ext. 4215

E-mail: iwasaki@tmig.or.jp

**Supplementary Table S1.** Study participants' responses to IAT items

| # | IAT items | Response |  |  |  |  |
| --- | --- | --- | --- | --- | --- | --- |
|  |  | 1 = Rarely | 2 = Occasionally | 3 = Frequently | 4 = Often | 5 = Always |
| 1 | How often do you find that you stay online longer than you intended? | 91 (5.8%) | 350 (22.4%) | 449 (28.7%) | 461 (29.5%) | 211 (13.5%) |
| 2 | How often do you neglect household chores to spend more time online? | 463 (29.6%) | 453 (29.0%) | 374 (23.9%) | 198 (12.7%) | 74 (4.7%) |
| 3 | How often do you prefer the excitement of the internet to intimacy with your partner? | 862 (55.2%) | 427 (27.3%) | 185 (11.8%) | 65 (4.2%) | 23 (1.5%) |
| 4 | How often do you form new relationships with fellow online users? | 798 (51.1%) | 330 (21.1%) | 250 (16.0%) | 135 (8.6%) | 49 (3.1%) |
| 5 | How often do others in your life complain to you about the amount of time you spend online? | 438 (28.0%) | 503 (32.2%) | 396 (25.4%) | 176 (11.3%) | 49 (3.1%) |
| 6 | How often do your grades or schoolwork suffer because of the amount of time you spend online? | 490 (31.4%) | 501 (32.1%) | 340 (21.8%) | 168 (10.8%) | 63 (4.0%) |
| 7 | How often do you check your email before something else that you need to do? | 386 (24.7%) | 495 (31.7%) | 369 (23.6%) | 222 (14.2%) | 90 (5.8%) |
| 8 | How often does your performance or productivity suffer because of the internet? | 388 (24.8%) | 462 (29.6%) | 301 (19.3%) | 268 (17.2%) | 143 (9.2%) |
| 9 | How often do you become defensive or secretive when anyone asks you what you do online? | 998 (63.9%) | 334 (21.4%) | 171 (10.9%) | 40 (2.6%) | 19 (1.2%) |
| 10 | How often do you block out disturbing thoughts about your life with soothing thoughts of the internet? | 544 (34.8%) | 485 (31.0%) | 283 (18.1%) | 180 (11.5%) | 70 (4.5%) |
| 11 | How often do you find yourself anticipating when you will go online again? | 459 (29.4%) | 432 (27.7%) | 334 (21.4%) | 229 (14.7%) | 108 (6.9%) |
| 12 | How often do you fear that life without the internet would be boring, empty, and joyless? | 582 (37.3%) | 437 (28.0%) | 302 (19.3%) | 164 (10.5%) | 77 (4.9%) |
| 13 | How often do you snap, yell, or act annoyed if someone bothers you while you are online? | 798 (51.1%) | 455 (29.1%) | 211 (13.5%) | 72 (4.6%) | 26 (1.7%) |
| 14 | How often do you lose sleep due to being online? | 478 (30.6%) | 452 (28.9%) | 297 (19.0%) | 234 (15.0%) | 101 (6.5%) |
| 15 | How often do you feel preoccupied with the internet when off-line or fantasize about being online? | 1,062 (68.0%) | 291 (18.6%) | 127 (8.1%) | 62 (4.0%) | 20 (1.3%) |
| 16 | How often do you find yourself saying "just a few more minutes" when online? | 483 (30.9%) | 417 (26.7%) | 304 (19.5%) | 247 (15.8%) | 111 (7.1%) |
| 17 | How often do you try to cut down the amount of time you spend online and fail? | 489 (31.3%) | 493 (31.6%) | 291 (18.6%) | 214 (13.7%) | 75 (4.8%) |
| 18 | How often do you try to hide how long you've been online? | 1,052 (67.3%) | 305 (19.5%) | 136 (8.7%) | 58 (3.7%) | 11 (0.7%) |
| 19 | How often do you choose to spend more time online over going out with others? | 1,056 (67.6%) | 286 (18.3%) | 133 (8.5%) | 60 (3.8%) | 27 (1.7%) |
| 20 | How often do you feel depressed, moody, or nervous when you are off-line, which goes away once you are back online? | 1,192 (76.3%) | 256 (16.4%) | 78 (5.0%) | 28 (1.8%) | 8 (0.5%) |

IAT=Young’s Internet Addiction Test

**Supplementary Table S2.** Mediation of the associations between internet addiction defined using different IAT cutoff points and dental caries by unhealthy lifestyle behaviors (N=1562)

|  | Outcome=DMFT | | |
| --- | --- | --- | --- |
|  | Mediator=ULBI | | |
| Exposure=Internet addiction (1=present [IAT score ≥40], 0=absent [IAT score <40]) | IRR^*^ | 95% CI | p value |
| Natural direct effect | 1.13 | 0.94–1.30 | 0.32 |
| Natural indirect effect | 1.04 | 1.01–1.07 | <0.01 |
| Total effect | 1.17 | 0.97–1.35 | 0.10 |
| Proportion mediated | 24.8% | |  |

CI=confidence interval, DMFT=the number of decayed, missing, and filled permanent teeth, IAT=Young’s Internet Addiction Test, IRR=incidence rate ratio, ULBI=unhealthy lifestyle behavior index

^*^Adjusted for grade, sex, receiving professional tooth brushing instruction, use of fluoride toothpaste, fluoride application, regular dental visits, and overweight.

**Supplementary Table S3.** Mediation of the associations between internet addiction and the number of decayed permanent teeth by unhealthy lifestyle (N=1562)

|  | Outcome=DT | | |
| --- | --- | --- | --- |
|  | Mediator=ULBI | | |
| Exposure=Internet Addiction (1=present [IAT score ≥50], 0=absent [IAT score <50]) | IRR^*^ | 95% CI | p value |
| Natural direct effect | 0.67 | 0.42–1.01 | 0.24 |
| Natural indirect effect | 1.18 | 1.08–1.30 | <0.01 |
| Total effect | 0.79 | 0.51–1.15 | 0.41 |
| Proportion mediated | -^†^ | |  |

CI=confidence interval, DT = the number of decayed permanent teeth, IAT=Young’s Intern Addiction Test, IRR=incidence rate ratio, ULBI=unhealthy lifestyle behavior index

^*^Adjusted for grade, sex, receiving professional tooth brushing instruction, use of fluoride toothpaste, fluoride application, regular dental visits, and overweight.

^†^The direct and indirect effects have opposite directions. The proportion mediated cannot be calculated.
